# *Staphylococcus aureus* and Lipopolysaccharide Modulate Gene Expressions of Drug Transporters in Mouse Mammary Epithelial Cells Correlation to Inflammatory Biomarkers

**DOI:** 10.1371/journal.pone.0161346

**Published:** 2016-09-01

**Authors:** Yagmur Yagdiran, Jonas Tallkvist, Karin Artursson, Agneta Oskarsson

**Affiliations:** 1 Department of Biomedical Sciences and Veterinary Public Health, Swedish University of Agricultural Sciences, Uppsala, Sweden; 2 National Veterinary Institute, Uppsala, Sweden; National Institutes of Health, UNITED STATES

## Abstract

Inflammation in the mammary gland (mastitis) is the most common disease in dairy herds worldwide, often caused by the pathogens *Staphylococcus aureus (S*. *aureus)* and *Escherichia coli (E*. *coli)*. Little is known about the effects of mastitis on drug transporters and the impact on transporter-mediated excretion of drugs into milk. We used murine mammary epithelial HC11 cells, after lactogenic differentiation into a secreting phenotype, and studied gene expressions of ABC- and SLC- transporters after treatment of cells with *S*. *aureus* and lipopolysaccharide, an endotoxin secreted by *E*. *coli*. The studied transporters were Bcrp, Mdr1, Mrp1, Oatp1a5, Octn1 and Oct1. In addition, Csn2, the gene encoding β-casein, was analyzed. As biomarkers of the inflammatory response, gene expressions of the cytokines Il6 and Tnfα and the chemokine Cxcl2 were determined. Our results show that *S*. *aureus* and LPS treatment of cells, at non-cytotoxic concentrations, induced an up-regulation of *Mdr1* and of the inflammatory biomarkers, except that *Tnfα* was not affected by lipopolysaccharide. By simple regression analysis we could demonstrate statistically significant positive correlations between each of the transporters with each of the inflammatory biomarkers in cells treated with *S*. *aureus*. The coefficients of determination (R^2^) were 0.7–0.9 for all but one correlation. After treatment of cells with lipopolysaccharide, statistically significant correlations were only found between *Mdr1* and the two parameters *Cxcl2* and *Il6*. The expression of *Csn2* was up-regulated in cells treated with *S*. *aureus*, indicating that the secretory function of the cells was not impaired. The strong correlation in gene expressions between transporters and inflammatory biomarkers may suggest a co-regulation and that the transporters have a role in the transport of cytokines and chemokines. Our results demonstrate that transporters in mammary cells can be affected by infection, which may have an impact on transport of essential compounds and contaminants into milk.

## Introduction

Transporters are membrane proteins responsible for mediating in- and efflux transport of endogenous substrates, numerous drugs and other chemicals. [1–5]. ATP-binding cassette (ABC-) and Solute Carrier (SLC-) superfamilies are the two major classes of transporters. Individual members of these superfamilies, such as BCRP, MDR1, MRP1, OATP1A2 (the human orthologue of the mouse Oatp1a5), OCTN1 and OCT1 are expressed in apical and basolateral membranes of epithelial cells in the mammary gland with differential expression during the pregnancy-lactation cycle, as reported in rodents, cows and humans [6–9]. BCRP, which is highly expressed in the mammary gland epithelium during lactation, has a high impact on drug transport into milk [10–15].

Mastitis is the most common disease in dairy herds worldwide with multifactorial aetiology, including bacterial pathogens causing local pain and reduced milk synthesis [16, 17]. Both duration and severity of mastitis have been suggested to depend on interactions between inflammatory stimuli and the host immune response [18, 19]. Common pathogens causing mastitis are *Staphylococcus aureus (S*. *aureus)* and *Escherichia coli (E*. *coli)* [20]. Lipopolysaccharide (LPS) is an endotoxin, which is secreted by gram negative bacteria such as *E*. *coli* [21].

Bovine mastitis is characterized by a mammary gland inflammation involving action of numerous cytokines and chemokines. The factors involved differ depending on the infectious agent [22–24]. In general, the proinflammatory cytokines, including tumor necrosis factor alpha (TNFα), interleukin-1 (IL1), interleukin-6 (IL6) and interferon gamma (IFNγ) further stimulate the synthesis of other cytokines and chemokines that bind to receptors on epithelial membranes.

Emerging evidence has demonstrated that expression and function of drug transporters are modulated by inflammation [25–30]. Liver, intestine, kidney, blood-brain barrier and placenta are the main studied tissues and the modulation of transporter activity has been connected to the activity of proinflammatory cytokines, including IL1, IL6 and TNFα [29]. However, little is known about the effect of inflammation in the mammary gland on the expression of drug transporters, which could have an impact on excretion of drugs into milk and on efficacy of treatment with drugs, which are ligands to the transporters. Effects of bovine mastitis on milk secretion of drugs have been reported for flunixin, enrofloxacin, norfloxacin, carprofen and azithromycin [31–35]. However, the impact of drug transporters on milk excretion of the drugs was not investigated in these studies.

Cell models are important tools to understand carrier-mediated transport mechanisms and they should preferably exhibit functional and morphological properties of corresponding *in vivo* cell layers. HC11 cells are derived from mammary gland tissue of BALB/C mice during mid-gestation and can be differentiated into a secreting phenotype with increased expression of β-casein by treatment with lactogenic hormones [36–38]. We have previously characterized gene expressions of transporters in mammary gland of mice at different lactation stages and in HC11 cells. Gene expressions of *Csn2 (β-casein)*, *Bcrp*, *Mdr1*, *Mrp1*, *Oatp1a5*, *Octn1* and *Oct1* were altered during gestation and lactation in mice mammary glands and in HC11 cells the expression patterns were affected by differentiation [9, 39].

Our aim was to investigate the effect of *S*. *aureus* and LPS treatment of mammary epithelial cells on gene expression of transporters of ABC- and SLC-superfamilies. The proinflammatory cytokines *Il6* and *Tnfα* and chemokine *Cxcl2* were determined as biomarkers of the inflammatory reaction. We used secreting murine mammary epithelial HC11cells treated with *S*. *aureus* and LPS and demonstrated effects on gene expression of transporters and strong positive correlations between the drug transporters and the inflammatory biomarkers.

## Materials and Methods

### Reagents and chemicals

Roswell Park Memorial Institute (RPMI) 1640 basal medium, gentamicin, heat-inactivated fetal bovine serum (FBS) and 0.05% Trypsin-EDTA were obtained from Gibco, via Life Technologies (Stockholm, Sweden). Human insulin, epidermal growth factor (EGF), prolactin, hydrocortisone and lipopolysaccharide from *Escherichia coli* O111:B4 (LPS) were purchased from Sigma-Aldrich (Stockholm, Sweden). Nucleospin RNA purification kit was obtained from Macherey-Nagel via AH diagnostics (Solna, Sweden) and Quant-iT^™^ RiboGreen^®^RNA Assay Kit from ThermoFisher Scientific via Life Technologies (Stockholm, Sweden). One-tube QuantiTect^™^SYBR^®^Green RT-PCR Kit was purchased from Qiagen Nordic (Sollentuna, Sweden) and CellTiter 96^®^ AQ_ueos_ One Solution Reagent was obtained from Promega Biotech AB (Nacka, Sweden). PBS tablets pH 7.4 were purchased from Medicago (Uppsala, Sweden).

### Cell culture and differentiation of cells

The HC11 murine mammary epithelial cell line was a generous gift from Dr. Nancy Hynes (Friedrich Miescher Institute for Biomedical Research, Basel, Switzerland) [40] and used with the permission of Dr. Bernd Groner (Institute for Biomedical Research, Frankfurt, Germany). Cells were cultured (passage 8–15) in sterile filtered RPMI 1640 medium containing 10% heat-inactivated FBS, 5 mg/L insulin, 10 μg/L EGF and 50 mg/L gentamycin in polycarbonate flasks at 37°C in 5% CO_2_. Medium was changed routinely every 2 or 3 days and cells subcultured by trypsination every 3 or 4 days. To induce differentiation of the cells to a secreting phenotype they were first seeded at a density of 500 000 cells/well in 6 well plates and cultured to confluency. Six days post-confluency the cells were incubated in medium without EGF for 24 hours. Following this EGF depletion step differentiation of the cells was accomplished by culturing for an additional 72 hours in serum- and EGF- free medium containing 1 mg/L prolactin and 1μM hydrocortisone. Differentiation of the cells was assessed by measuring induction of β-casein *(Csn2)* gene expression as well as examination of cellular morphology as described previously [39].

### *S*. *aureus* isolation and determination of concentration

*S*. *aureus* pathogen strain Mas106 was isolated from a case of acute clinical bovine mastitis [41]. The *S*. *aureus* strain was cultured on 5% bovine blood agar plates at 37°C overnight. One inoculation loop (1 μl) of *S*. *aureus* containing approximately 1x10^9^ colony forming units (CFU) was diluted to 1 ml in cell culture medium without antibiotics. This bacterial solution was further diluted to the desired concentrations (1x10^8^ and 1x 10^6^ CFU/ml). To determine the actual concentration of *S*. *aureus*, each used bacterial dilution was inoculated on bovine blood agar plates. Agar plates were incubated at 37°C during 18 hours, bacterial colonies were counted and the concentration estimated.

### Cell treatment with *S*. *aureus* and LPS

HC11 cells were seeded in 6-well culture plates and differentiated as described above. The medium was replaced with antibiotic free medium 14–18 hours before treatment with *S*. *aureus* strain Mas106. All incubation steps were carried out at 37°C at 5% CO_2._ After removing the growth medium 3 ml of serum-free RPMI 1640 medium without antibiotics containing 1x10^6^ and 1x10^8^ CFU/ml of *S*. *aureus*, respectively, were added to the wells with differentiated cells and the cells were incubated for 2 hours. Control cells were incubated with cell culture medium without bacteria. After incubation, each well was washed twice with 5 ml of pre-warmed (at 37°C) PBS after which 3 ml of serum-free RPMI 1640 medium with 100 μg/ml gentamicin was added and plates were incubated for an additional 2 hours. Cells were then washed twice with 5 ml of pre-warmed PBS per well and 3 ml of serum-free RPMI 1640 medium without gentamicin was added. Plates were incubated for another 3 hours, washed twice with 5 ml of pre-warmed PBS per well and then lysed with RA1 buffer for RNA isolation. The incubation periods were optimized to allow the highest intracellular uptake of bacteria without visually affecting cell viability. The experiment was repeated and in each experiment 6 wells were used per concentration.

Treatment of differentiated HC11 cells with LPS was performed in six wells at a concentration of 5 μg/ml in serum-free RPMI 1640 medium for 24 hours. Six control wells were treated with only medium. After treatment cells were washed with 3 ml PBS and then lysed with RA1 buffer for RNA isolation. RA1 lysates were stored at -70°C prior to isolation of RNA and gene expression analyzes as described below.

### Cell Viability Test

Prior to the treatment with *S*. *aureus* and LPS the cytotoxic effect was studied using a MTS-based colorimetric assay (Promega Corporation, Madison, WI, USA) in which mitochondrial activity in viable cells transforms tetrazolium compound into colored formazan. A total of 17 000 cells/well were seeded into 96-well plates in a volume of 100 μl, cultured and differentiated as described above. Cells were tested with 100 μl of either *S*. *aureus* suspensions (1 x 10^4^, 10^5^, 10^6^, 10^7^ 10^8^ CFU/ml) or vehicle controls as described above. After incubation with *S*. *aureus* or LPS, 20 μl CellTiter 96^®^ AQ_ueos_ One Solution Reagent was added to each well, according to MTS manufacturer’s instructions. After 1 hour of incubation at 37°C absorbance was measured at 490 nm using a Wallac Victor^2^1420 microplate reader (Perkin-Elmer, Massachusetts, USA). Cell viability was assessed by comparing mean absorbance values from bacteria or LPS treated cells and vehicle controls based on six replicates. Six wells were used per concentration and for controls.

### Isolation of total RNA and RT-qPCR analysis

Total RNA from cells was isolated by using the NucleoSpin RNA kit containing DNAse I as recommended by the manufacturer. To check the integrity of the RNA the 28 S and 18 S ribosomal RNA bands were examined by UV-visualization following agarose gel electrophoresis. Quantification of the RNA was performed with the RNA specific Quant-iT RiboGreen protocol as described by the manufacturer.

Gene specific intron spanning primers to murine *Cxcl2* were forward: 5’-TCCAGAGCTTGAGTGTGACG-3 and reverse: 5’-CTTTGGTTCTTCCGTTGAGG-3; *Il6* forward: 5’-AGTTGCCTTCTTGGGACTGA-3 and reverse: 5’- TCCACGATTTCCCAGAGAAC-3; *Tnfα* forward: 5’-AGCCCCCAGTCTGTATCCTT-3 and reverse: 5’-CTCCCTTTGCAGAACTCAGG-3 and designed by the use of University of California Santa Cruz (UCSC) Genome Browser and Primer3 software. The primers were synthetized by Cybergene (Huddinge, Sweden). Sequences of the other murine primers used are reported in [9].

Quantitative gene expression was examined by RT-qPCR using a Rotor-Gene 3000 (Corbett Research, Mortlake, Australia) by applying the One-tube QuantiTect^™^SYBR^®^Green RT-PCR Kit, according to the manufacturer’s recommendations. Murine mammary tissue was used as positive amplification controls and all primer-pairs for the target genes tested on RNA isolated from these tissues generated specific RT-PCR products with anticipated amplicon sizes and single melting curve peaks. Final primer concentration for all target genes was 0.4 μM and 75 or 150 ng total RNA was used as template in 12.5 μl RT-qPCR reactions. Non-template controls served as blanks and melt curve analysis was performed for each sample to check the specificity of the obtained PCR products. Expressions of target genes were normalized to the geometric average expression of three appropriate reference genes [42]. We used the three reference genes, recommended for normalization of RT-qPCR data in gene expression studies of mouse mammary gland: hypoxanthine-guanine phosphoribosyltransferase (*Hprt*), ribosomal protein L13A (*Rpl13a*) and glyceraldehyde 3-phosphate dehydrogenase (*Gapdh*) [43]. Relative quantification of mRNA expressions was performed by comparing the quantification cycle (Cq) between the tissues and treatment groups of cells according to the 2^-(ΔΔCq)^–method [44]. Cq cycle 35 was used as cut-off for limit of detection of gene expression. Fold differences were calculated setting vehicle controls to one.

### Statistical Analysis

Statistical analyses were performed by using Minitab 16 software. The results were analyzed by simple linear regression to assess correlation between transporters and inflammatory biomarkers and by Kruskal-Wallis to detect any significant differences among the various treatment groups, followed by Mann-Whitney to examine statistically significant differences between two groups. The level of significance was set at P < 0.05.

## Results

### Cell viability

**Fig 1:**
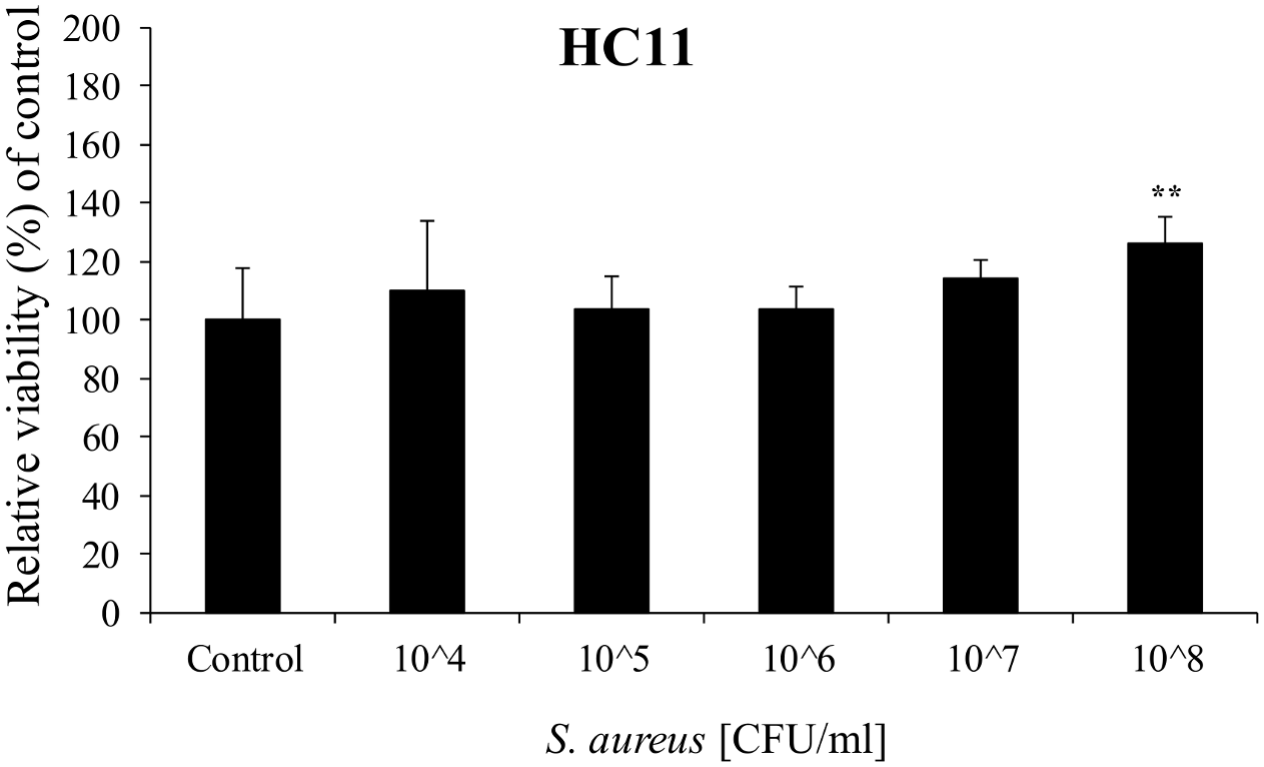
Cell viability in HC11 cells treated with *S*. *aureus* for 2 hours. Values are means ± SD; n = 6. Statistically significant differences compared to vehicle controls ** p < 0.01.

Cell viability after bacterial or LPS treatment of cells was assessed by MTS. No decrease in cell viability was observed after 2 hours incubation with *S*. *aureus* up to 1x10^8^ CFU/ml (Fig 1). A statistically significant increase in cell viability was observed at 1x10^8^ CFU/ml to 125% of control levels. Treatment of cells with 5 μg/ml LPS for 24 hours did not cause any effect on cell viability (98% ± 12) compared to controls (100% ± 8%) (mean ± S.D.; n = 6).

### Gene expressions in HC11 cells

**Fig 2:**
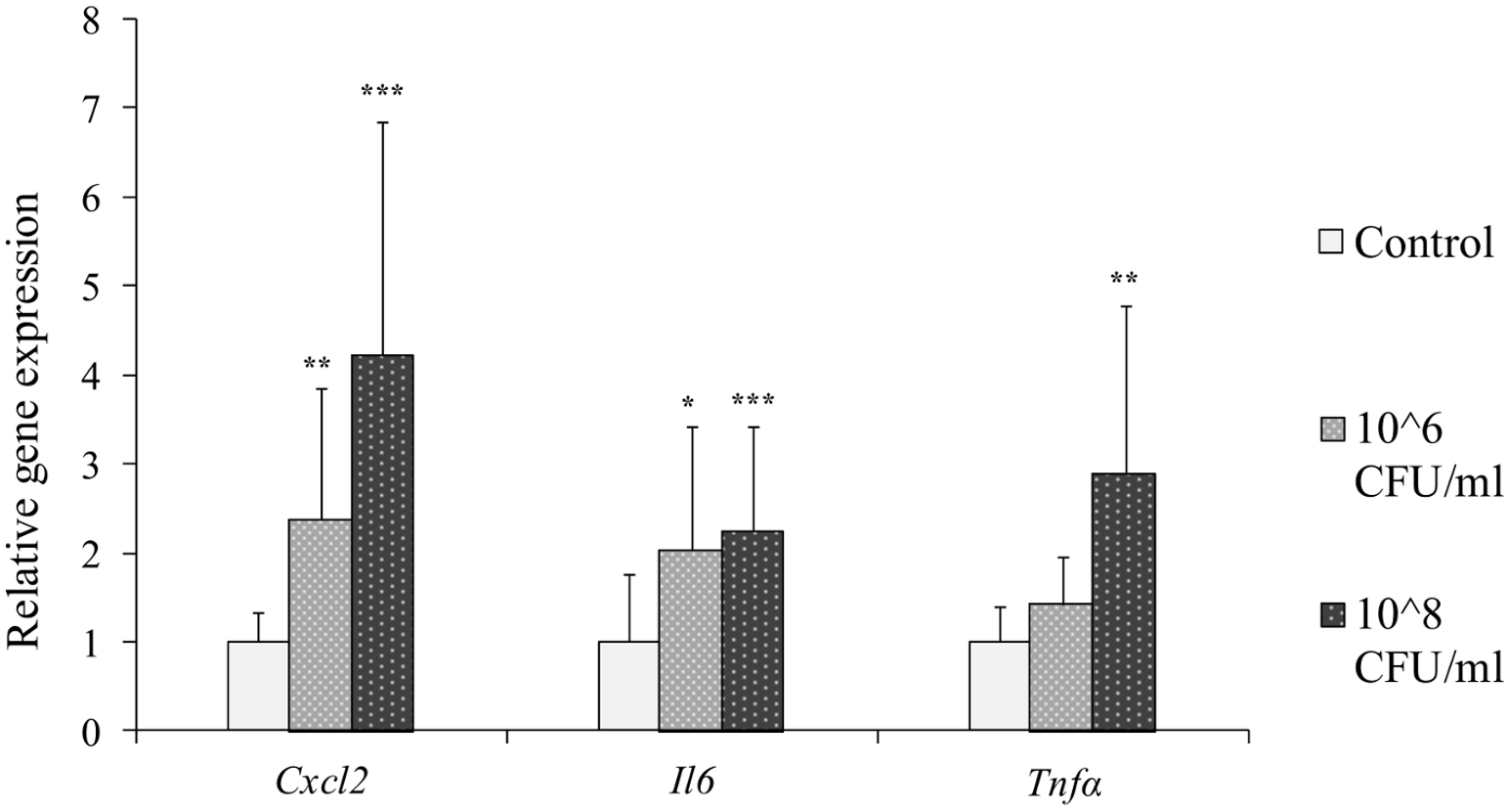
Relative gene expression of inflammatory biomarkers in HC11 cells treated with *S*. *aureus*. Differentiated HC11 cells were treated with *S*. *aureus* for 2 hours. Gene expressions of the inflammatory biomarkers *Cxcl2*, *Il6 and Tnfα* were determined. Normalized gene expressions are presented as means ± SD; n = 12 pooled from 2 separate experiments. Statistically significant differences compared to vehicle controls *p < 0.05; **p < 0.01, *** p < 0.001.

**Fig 3:**
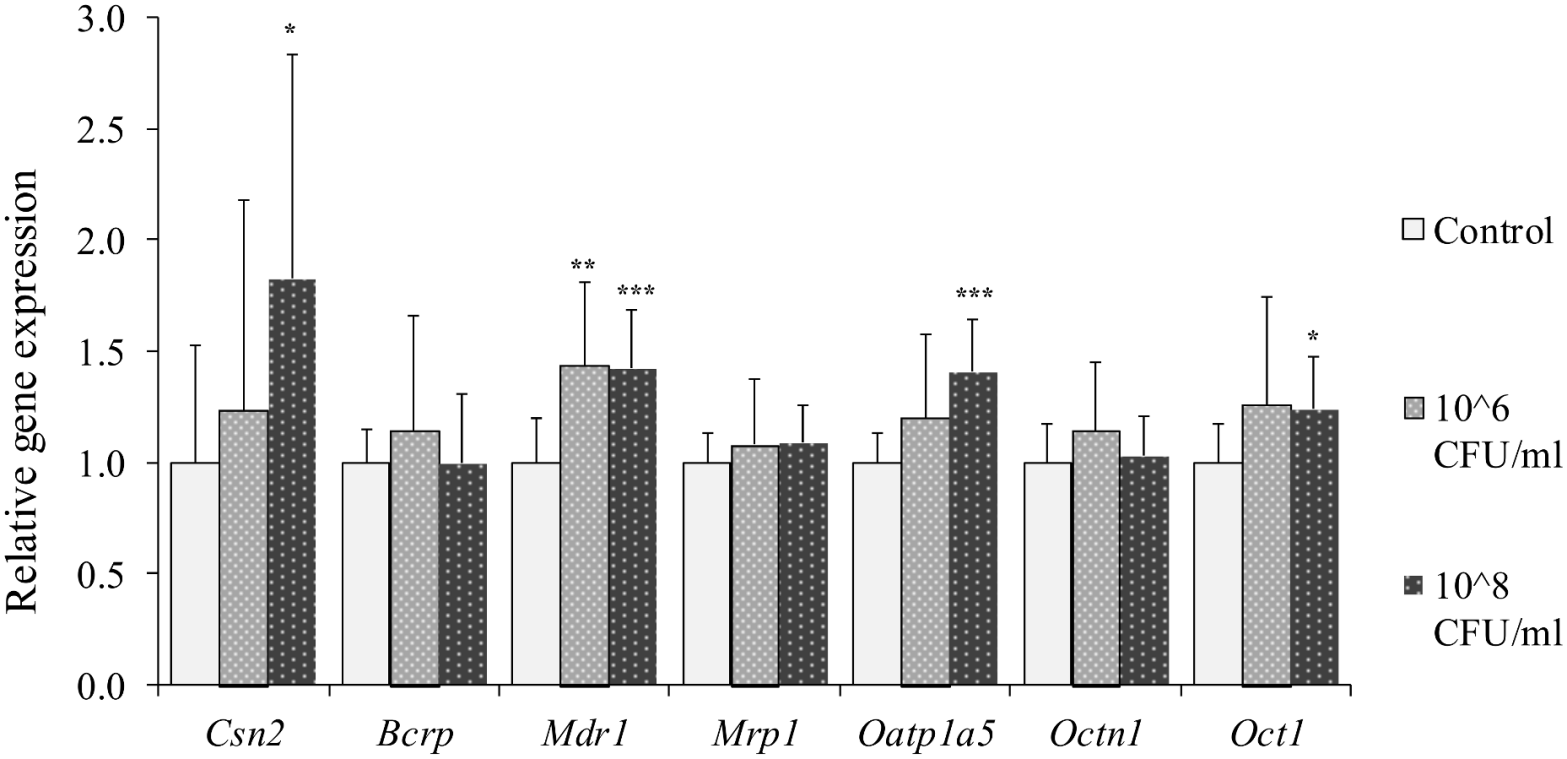
Relative gene expression of *Csn2* and transporters in HC11 cells treated with *S*. *aureus*. Differentiated HC11 cells were treated with *S*. *aureus* for 2 hours. Expressions of the genes encoding β-casein, *Csn2*, and the transporters *Bcrp*, *Mdr1*, *Mrp1*, *Oatp1a5*, *Octn1* and *Oct1* were determined. Normalized gene expressions are presented as means ± SD; n = 12 pooled from 2 separate experiments. Statistically significant differences compared to vehicle controls *p < 0.05; **p < 0.01, *** p < 0.001.

HC11 cells were treated with *S*. *aureus* at concentrations of 1x10^6^ and 1x10^8^ CFU/ml and expressions of genes encoding inflammatory biomarkers and β-casein and drug transporters were analyzed. The inflammatory biomarkers *Cxcl2* and *Il6* were statistically significantly up-regulated in cells after treatment with both concentrations *of S*. *aureus*, while *Tnfα* was significantly induced by *S*. *aureus* treatment only at the highest concentration (Fig 2). *S*. *aureus* treatment of cells resulted in an up-regulation of *Csn2*, *Mdr1*, *Oatp1a5* and *Oct1* at the highest concentration (Fig 3). *Mdr1* was up-regulated also at the lower bacteria concentration. No statistically significant differences were observed for gene expressions of *Bcrp*, *Mrp1* and *Octn1* after *S*. *aureus* challenge (Fig 3).

**Fig 4:**
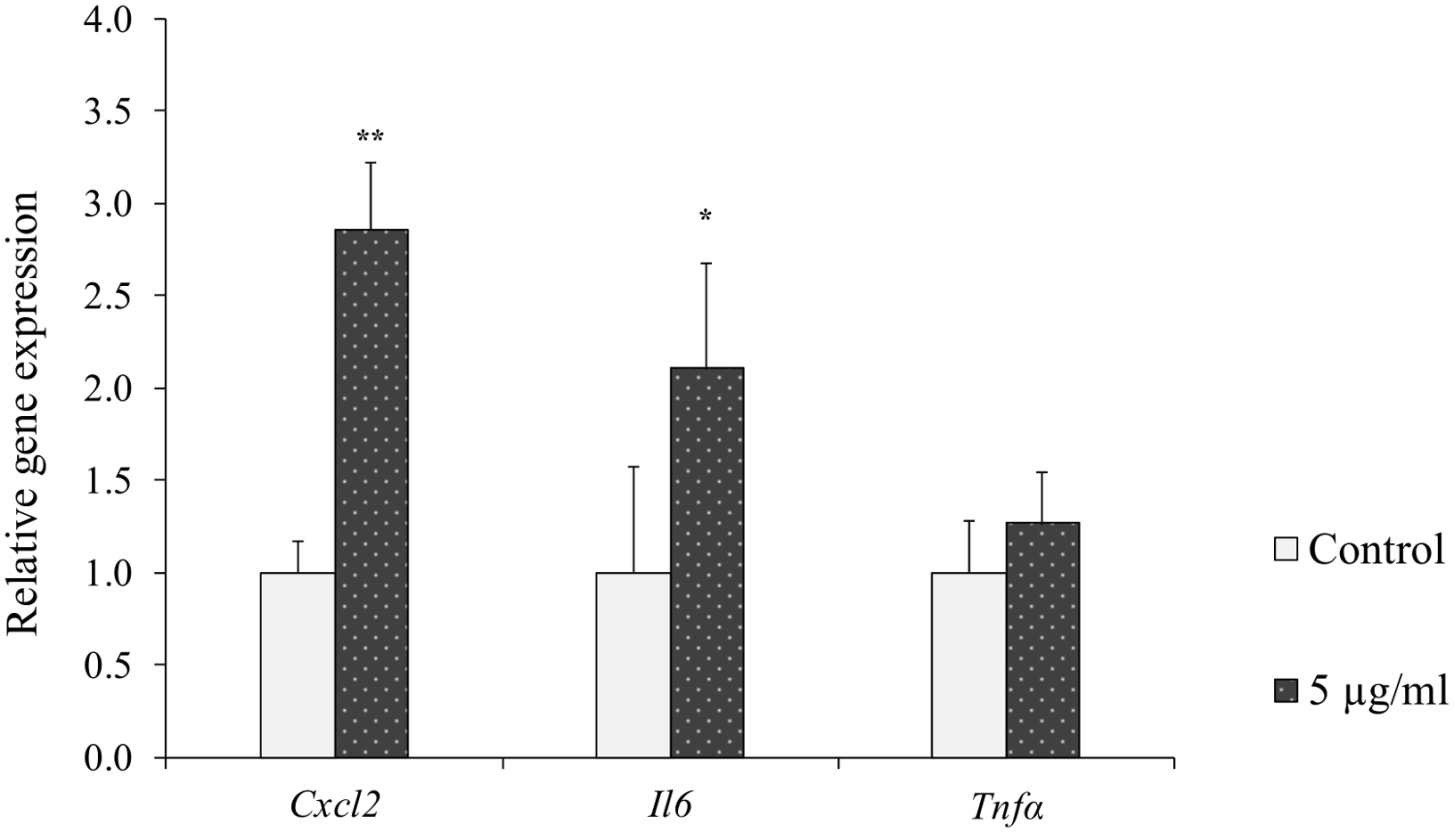
Relative gene expression of inflammatory biomarkers in HC11 cells treated with LPS. Differentiated HC11 cells were treated with LPS for 24 hours. Gene expressions of the inflammatory biomarkers *Cxcl2*, *Il6 and Tnfα* were determined. Normalized gene expressions are presented as means ± SD; n = 6. Statistically significant differences compared to vehicle controls *p < 0.05; **p < 0.01.

**Fig 5:**
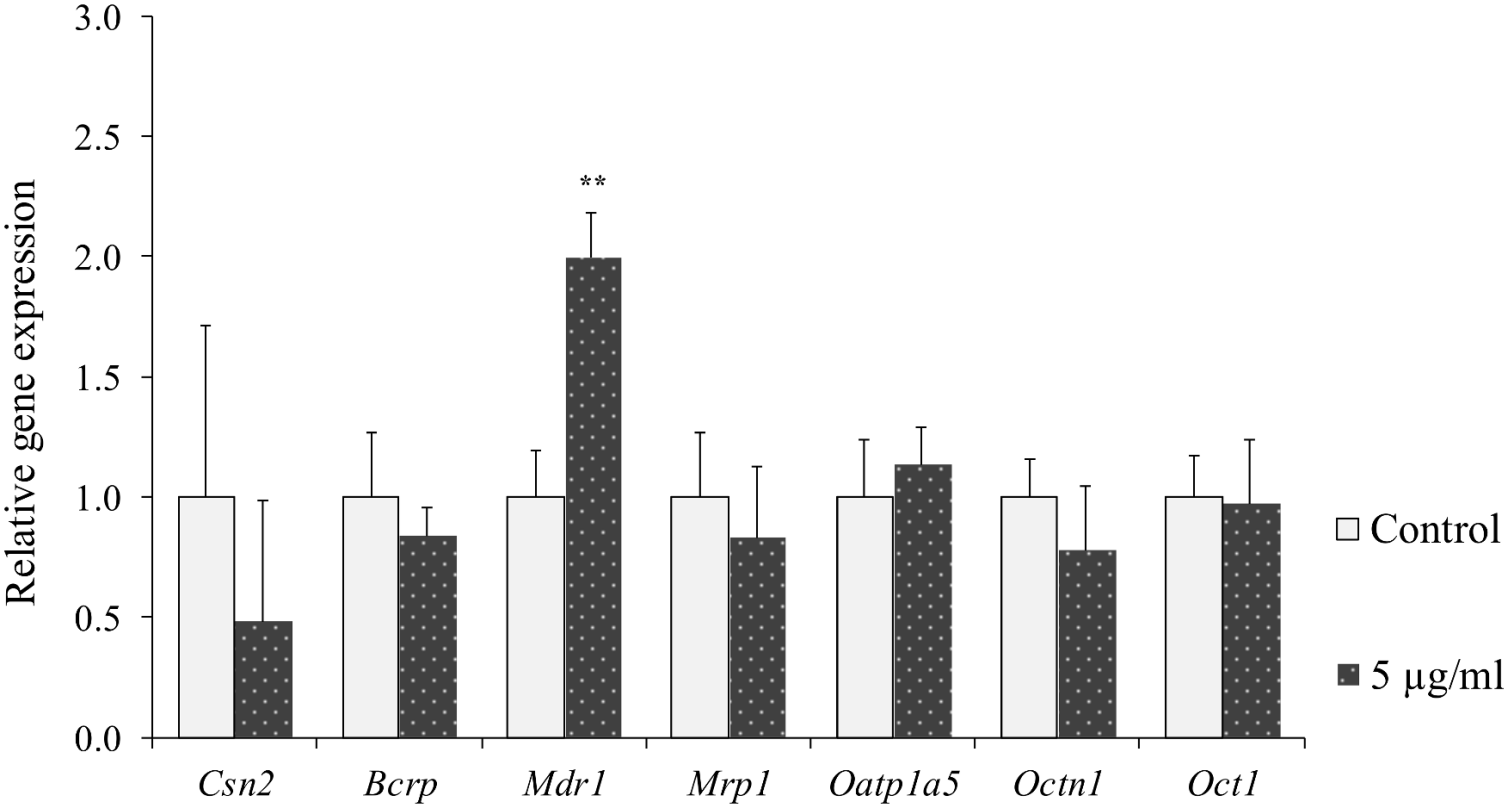
Relative gene expression of *Csn2* and transporters in HC11 cells treated with LPS. Differentiated HC11 cells were treated with LPS for 24 hours. Expressions of the genes encoding β-casein, *Csn2*, and the transporters *Bcrp*, *Mdr1*, *Mrp1*, *Oatp1a5*, *Octn1* and *Oct1* were determined. Normalized gene expressions are presented as means ± SD; n = 6. Statistically significant differences compared to vehicle controls **p < 0.01.

In cells treated with 5 μg/ml LPS statistically significant up-regulation of *Cxcl2* and *Il6* expression was observed as compared to untreated controls, while no statistically significant difference was observed on *Tnfα* expression (Fig 4). Treatment of cells with LPS resulted in statistically significant up-regulation of *Mdr1* expression compared to controls (Fig 5). No statistically significant difference due to LPS treatment was observed on any of the other genes tested (Fig 5).

**Fig 6:**
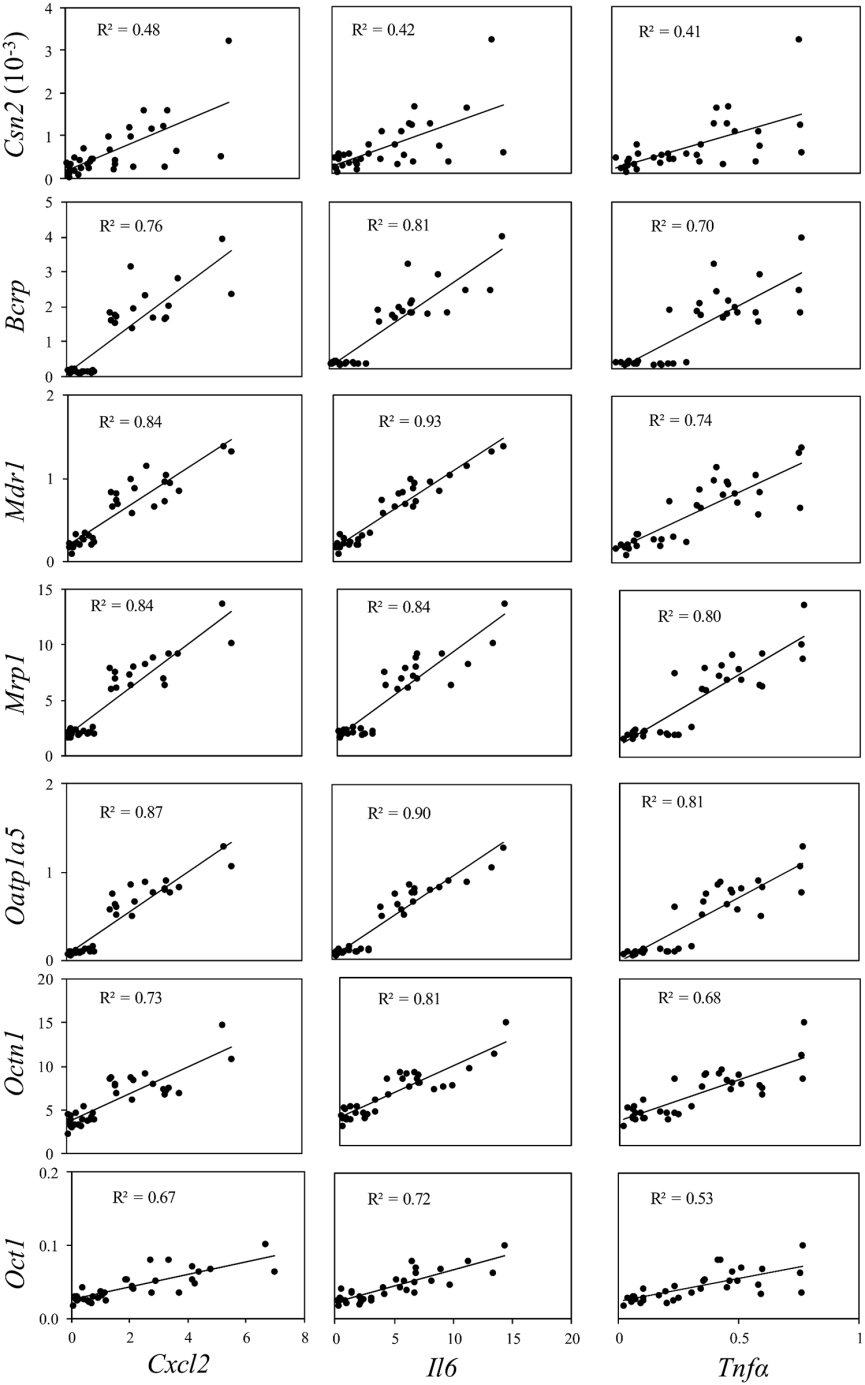
Regression plots with individual data from 2 hours treatment of HC11 cells with *S*. *aureus*. Data from Figs 2 and 3 were analysed by simple linear regression analysis of dependent variable gene expression of transporters (*Bcrp*, *Mdr1*, *Mrp1*, *Oatp1a5*, *Octn1* and *Oct1*) or *Csn2* and independent variable gene expression of inflammatory biomarkers (*Cxcl2*, *Il6 and Tnfα*). p < 0.001 in all regressions.

**Fig 7:**
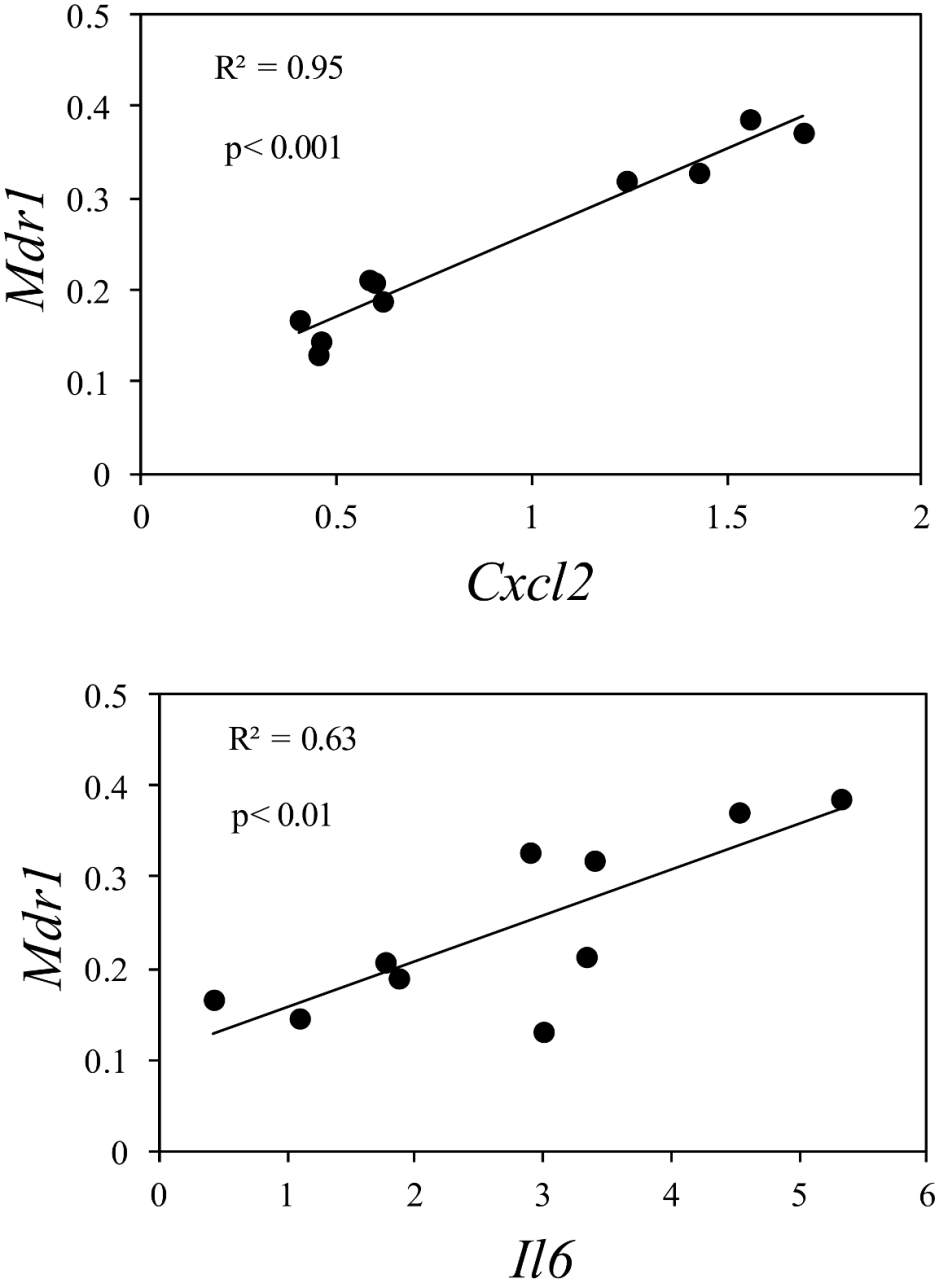
Regression plots with individual data from 24 hours treatment of differentiated HC11 cells with LPS. Data from Figs 4 and 5 were analysed by simple linear regression analysis of dependent variable gene expression of *Mdr1* and independent variable gene expression of the inflammatory biomarkers *Cxcl2* (p < 0.001) and *Il6* (p < 0.01).

To examine the correlation between gene expressions of transporters and the inflammatory biomarkers using all individual data, a simple linear regression analysis was performed for the results from *S*. *aureus* and LPS treatments (Figs 6 and 7). In addition, correlations between C*sn*2 and the inflammatory biomarkers were tested. In HC11 cells treated with *S*. *aureus* significant positive correlations were detected between each of the transporters with each of the inflammation biomarkers (p < 0.001) (Fig 6). With one exception, between *Oct1* and *Tnfα*, all coefficients of determination (R^2^) were between 0.7 and 0.9, indicating that ≥ 70% of the variation in expression of transporters can be explained by inflammatory biomarkers. Treatment of cells with *S*. *aureus* resulted in a significant positive correlation also between C*sn*2 and the biomarkers with R^2^ values of 0.4–0.5 (Fig 6). In cells treated with LPS only two significant correlations were found. Both were for *Mdr*1, one with *Cxcl2* and one with *Il6* (Fig 7).

## Discussion

Little is known about the effect of inflammation in the mammary gland on the expression of drug transporters. This is surprising, as mastitis is a common pathological condition in dairy cows, varying from severe clinical to subclinical forms. Milk secretion of drugs has been demonstrated to be affected by mastitis in cows [31–35], although the role of drug transporters as a mechanism behind these effects has not been investigated to our knowledge.

In the present study we found a statistically significant up-regulation of *Mdr1*, the gene encoding the efflux transporter MDR1 (Pg-p, ABCB1), after treatment of differentiated murine HC11 mammary cells with non-cytotoxic concentrations of *S*. *aureus* and LPS. Furthermore, treatment of cells by *S*. *aureus* up-regulated the gene expressions of *Oatp1a5* and *Oct1*, as well as *Csn2*, the gene encoding β-casein, at the highest concentration of *S*. *aureus*. *Bcrp*, *Mrp1* and *Octn1* were not affected by the treatments. We have recently demonstrated, in the same cell line, that the fungicide prochloraz induces gene expression of *Mdr1* as well as protein expression and function of MDR1, measured as decreased digoxin accumulation (Yagdiran et al, 2016). In the current investigation protein expression and function was not studied, but it may be plausible that the induced gene expression of *Mdr1* by *S*. *aureus* and LPS is also associated with induced protein induction and function of MDR1. Furthermore, Al-Bataineh et al [45] found a strong correlation between gene and protein expression of MDR1 and function of the transporter in a bovine mammary epithelial cell line, BME-UV, treated with TNFα.

As biomarkers of inflammation we used gene expressions of the chemokine *Cxcl2* and the proinflammatory cytokines *Il6* and *Tnfa* and found a concentration dependent response in the biomarkers after treatment of cells with *S*. *aureus*. A similar response was observed after treatment of cells with LPS with an up-regulation of *Cxcl2* and *Il6*, while *Tnfa* did not increase after the LPS treatment.

By simple linear regression analysis, using all individual data on transporters and inflammatory biomarkers from *S*. *aureus* treated cells, we were able to demonstrate statistically significant positive correlations between the expressions of each of the transporters with the expression of each of the biomarkers. These correlations could well have been overlooked if data had been analyzed just by comparing the dose groups. The correlations were strong, varying in coefficient of determination (R^2^) between 0.53 for the correlation between *Oct1* and *Tnfα* to 0.93 for the correlation between *Mdr1* and *Il6*. Thus, 93% of the variation in *Mdr1* can be explained by *Il6*. The strong correlations between transporters and biomarkers may indicate that the two parameters are co-regulated, acting in the same pathways and/or that the cytokines and the chemokine are mediators in regulating the expression of the transporters in the mammary epithelial cells. Indeed, a cytokine mediated regulation of anion transporters in endotoxin-induced inflammation of murine liver *in vivo* and *in vitro* has been suggested [46]. Possible mechanisms for regulation of MDR1 by cytokines have been discussed by Liptrott and Owen [47] and involve direct action of cytokine (IL-6) on genes encoding transporters via immune-related transcription factors, such as activator protein-1 (AP-1). It may also involve TNFα activation of NF-κB, which then activates target genes. These suggestions are supported by the finding that MDR1 gene and protein induction by vinblastine in Caco-2 cells was mediated by the activation of AP-1 and NF-κB [48]. The function of the transporters in the inflammation response is not known, but there is evidence that efflux transporters have a potential role in the transmembrane flux of cytokines. Drach et al. [49] demonstrated that P-glycoprotein mediated the transport of IL2, IL4 and IFNγin T lymphocytes, which was further supported by Pawlik et al. [50], who reported that P-glycoprotein inhibitors suppressed the release of IL2, IL4, IFNγ and TNFα in peripheral blood mononuclear cells stimulated with phytohaemagglutinin. In addition, it is well known that MRP1 is a transporter for cysteinyl-leukotriene C4, a lipid mediator of inflammation [51].

Regression analysis of the data from LPS treatment of cells resulted in only two statistically significant correlations between transporters and biomarkers, both on *Mdr1*¸ one with *Cxcl2* and one with *Il6*. One reason for not being able to demonstrate potential correlations between the other parameters may be the low number of data points and the more narrow range in expression levels of the biomarkers after LPS treatment, compared with *S*. *aureus* treatment of cells. However, it cannot be excluded that there is a real difference in gene expression response of transporters between cells treated with *S*. *aureus* and LPS, as has previously been demonstrated for cytokines [23, 52, 53]. A correlation between *Mdr1* and *Tnfa* is not expected, due to the fact that *Tnfa* was not up-regulated by LPS. Lack of effect on *Tnfa* after LPS treatment of HC11 cells has been reported previously by Li et al. [54], who showed that LPS treatment induced lactoferrin, which blocked *Tnfa*, but did not affect *Il6* gene expression in the cells.

The expression of *Csn2* was up-regulated in cells after treatment with *S*. *aureus*, but not after LPS treatment. When HC11 cells are treated with prolactin, cortisol and insulin, the cells adopt a secretory phenotype resembling mammary epithelial cells in the lactating mammary gland, including increased β-casein gene expression [37, 38]. The reason for an upregulation of *Csn2* by *S*. *aureus* is not known, but there are no indications that the secretory phenotype is lost by the *S*. *aureus* treatment. It may be suggested that our cell model with *S*. *aureus* is mimicking subclinical mastitis, when milk production is not affected to a substantial extent. Thus, the conditions used in our study with *S*. *aureus* did not result in any decrease in cell viability or *Csn2* expression, while inflammation biomarkers and transporters were affected. Furthermore, we treated cells with the infectious agent S. aureus or the endotoxin LPS to get an endogenous production of cytokines by the cells, which presumably is a more relevant exposure model for mastitis than treating directly with recombinant cytokines.

Previous studies on effects of inflammation on transporters, both *in vivo* and *in vitro*, have used treatment of LPS or cytokines and reported in most cases down-regulation of expression and function of the transporters MDR1, MRPs and OATPs in the liver [46, 55–59]. However, there are also studies showing up-regulation of MDR1 in the rat liver [60], rat kidney proximal tubule cells [61], and rat brain capillaries [62]. Furthermore, inflammation-induced changes of transporters in immune cells are reported [47, 63]. Upregulation of gene and protein expression and function of MDR1 were reported in human blood mononuclear cells after treatment with cytokines [64] and gene expression of MDR1 was upregulated in the monocytic cell line THP-1 after infection with *Listeria monocytogenes* [65]. We have only found two studies in mammary epithelial cells on drug transporters after treatment with cytokines and both reported up-regulation of transporters. Increased expression and function of MDR1was demonstrated in bovine mammary epithelial (BMV-UV) cells treated with TNFα [45]. Mosaffa et al. [66] reported increased expression of BCRP in breast carcinoma (MCF-7) cells after treatment with TNFα. To our knowledge there are no previous reports demonstrating correlations between transporters and proinflammatory cytokines/chemokine.

Increased expression and function of efflux transporters due to mastitis could have an impact not only on the transport of essential endogenous compounds into milk, but also cause increased concentrations in milk of drugs and other chemicals, which are substrates to the transporters. In addition, an up-regulation of efflux transporters may decrease the efficacy of mastitis therapy, when using drugs, which are substrates of the transporters. Our results showing a strong correlation in gene expressions between transporters and inflammatory biomarkers may suggest a co-regulation and that the efflux transporters may have a role in the transmembrane transport of cytokines and chemokines.
