# Correction: *Staphylococcus aureus* and Lipopolysaccharide Modulate Gene Expressions of Drug Transporters in Mouse Mammary Epithelial Cells Correlation to Inflammatory Biomarkers

**DOI:** 10.1371/journal.pone.0168929

**Published:** 2016-12-16

**Authors:** The PLOS ONE Staff

There is an error in the article title. The publisher apologizes for the error. The correct title is: *Staphylococcus aureus* and Lipopolysaccharide Modulate Gene Expressions of Drug Transporters in Mouse Mammary Epithelial Cells–Correlation to Inflammatory Biomarkers.

The correct citation is Yagdiran Y, Tallkvist J, Artursson K, Oskarsson A (2016) *Staphylococcus aureus* and Lipopolysaccharide Modulate Gene Expressions of Drug Transporters in Mouse Mammary Epithelial Cells–Correlation to Inflammatory Biomarkers. PLoS ONE 11(9): e0161346. doi:10.1371/journal.pone.0161346

## References

[1] Yagdiran Y, Tallkvist J, Artursson K, Oskarsson A (2016) *Staphylococcus aureus* and Lipopolysaccharide Modulate Gene Expressions of Drug Transporters in Mouse Mammary Epithelial Cells Correlation to Inflammatory Biomarkers. PLoS ONE 11(9): e0161346 doi: 10.1371/journal.pone.0161346 27584666 10.1371/journal.pone.0161346 PMC5008833

